# Cubosomal Lipid Formulation for Combination Cancer Treatment: Delivery of a Chemotherapeutic Agent and Complexed α-Particle Emitter ^213^Bi

**DOI:** 10.1021/acs.molpharmaceut.2c00182

**Published:** 2022-07-18

**Authors:** Adrianna Cytryniak, Kinga Żelechowska-Matysiak, Ewa Nazaruk, Renata Bilewicz, Rafał Walczak, Emilia Majka, Adam Mames, Frank Bruchertseifer, Alfred Morgenstern, Aleksander Bilewicz, Agnieszka Majkowska-Pilip

**Affiliations:** † Faculty of Chemistry, University of Warsaw, Pasteura 1, 02-093 Warsaw, Poland; ‡ Centre of Radiochemistry and Nuclear Chemistry, Institute of Nuclear Chemistry and Technology, Dorodna 16, 03-195 Warsaw, Poland; § Institute of Physical Chemistry, Polish Academy of Sciences, Kasprzaka 44/52, 01-224 Warsaw, Poland; ∥ Directorate for Nuclear Safety and Security, European Commission, Joint Research Centre, Postfach 2340, 76125 Karlsruhe, Germany

**Keywords:** cubosomes, α-therapy, ^213^Bi radionuclide, doxorubicin, cytotoxicity, cancer cells

## Abstract

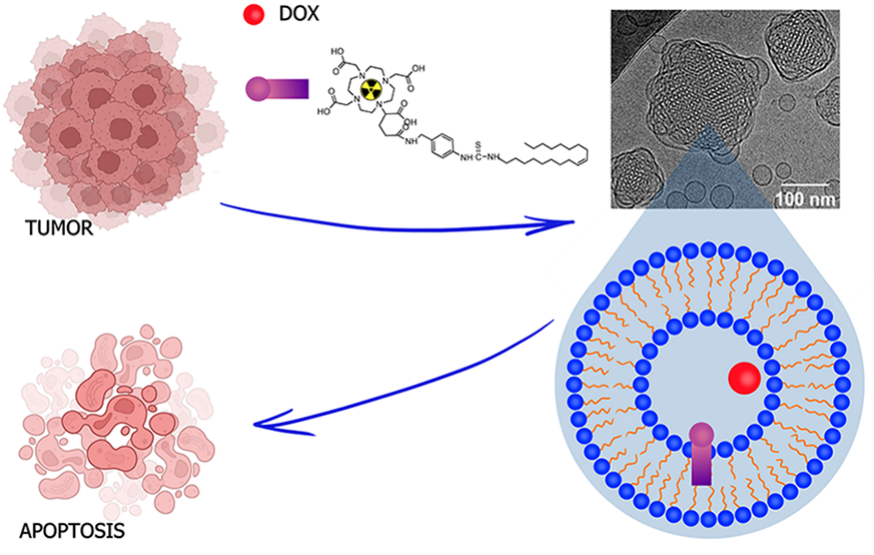

Here, we propose tailored lipid liquid-crystalline carriers (cubosomes), which incorporate an anticancer drug (doxorubicin) and complexed short-lived α-emitter (bismuth-213), as a strategy to obtain more effective action toward the cancer cells. Cubosomes were formulated with doxorubicin (DOX) and an amphiphilic ligand (DOTAGA-OA), which forms stable complexes with ^213^Bi radionuclide. The behavior of DOX incorporated into the carrier together with the chelating agent was investigated, and the drug liberation profile was determined. The experiments revealed that the presence of the DOTAGA-OA ligand affects the activity of DOX when they are incorporated into the same carrier. This unexpected influence was explained based on the results of release studies, which proved the contribution of electrostatics in molecular interactions between the positively charged DOX and negatively charged DOTAGA-OA in acidic and neutral solutions. A significant decrease in the viability of HeLa cancer cells was achieved using sequential cell exposure: first to the radiolabeled cubosomes containing ^213^Bi complex and next to DOX-doped cubosomes. Therefore, the sequential procedure for the delivery of both drugs encapsulated in cubosomes is suggested for further biological and *in vivo* studies.

## 1 Introduction

Surgery, chemotherapy, and external radiotherapy are the most common therapies currently used for cancer treatment. However, for addressing the spread of cancers, chemotherapy is the most commonly used therapy. One of the most widespread chemotherapeutic drugs for the treatment of solid tumors and malignancies, malignant lymphoma, and acute leukemia is doxorubicin (DOX). However, like other chemotherapeutic agents, there are serious limiting factors for the clinical applications of DOX, such as dose-dependent irreversible cardiotoxicity and poor specificity.^1,2^ These factors lead to the administration of suboptimal doses of DOX. As a result, therapeutic failure and development of drug resistance are observed.

Another important strategy in cancer treatment is internal radiation therapy—endoradiotherapy,^3^ which represents an alternative therapeutic method and has advantageous features compared to chemotherapy and external radiation therapies. Intelligent dose delivery concepts using small molecules, peptides, or antibodies as radionuclide carriers enable the selective accumulation of the radiation sources in the tumor lesion and reduction of radiation toxicity for the peripheral organs. Bioconjugates labeled with β^–^- or α-emitting radionuclides have found widespread use in cancer therapy.^4^^90^Y- and ^177^Lu-labeled peptides or small molecules, like PSMA-617, somatostatin, and bombesin analogues, show very promising results.^5,6^ DOTATATE, a drug labeled with ^177^Lu (Lutathera), was approved by the U.S. Food and Drug Administration (FDA) in 2018 for the treatment of certain neuroendocrine tumors. A challenge associated with β^–^-emitting targeted radionuclide therapies is the inherent toxicity caused by the death of normal healthy cells, resulting from crossfire radiation damage from the relatively long ranges of the β^–^ particles in the tissue.^7^ An emerging strategy is the use of targeted α-particle therapy.^8−10^ The use of α-particles in the targeted radionuclide therapy offers a beneficial alternative to the use of β-particles. α-particles deposit all of their energy within a much shorter range, equating to a few cell diameters (50–100 μm). As a result, targeted α-radiotherapy agents have great potential for the treatment of small and disseminated tumors. It also has possible therapeutic applications in the treatment of micrometastases and hematological malignancies consisting of individual, circulating neoplastic cells. Unlike β-particles, α-particles afford a very high relative biological efficacy and can destroy more cancer cells with less radioactivity. α-particles can induce significantly more DNA double-strand breaks in comparison to β-particles due to their high linear energy transfer. Vitally, the biological efficacy of α-particles is not hindered by cell cycle considerations and is not dependent on hypoxia.^11,12^

The major α-emitters used now for targeted radionuclide therapy are the following: ^223,224^Ra, ^211^At, ^212^Pb, ^226,227^Th, ^225^Ac, and ^212,213^Bi.^13^ Of them all, ^213^Bi proved to be a promising radionuclide for cancer treatment in clinical investigations. ^213^Bi is a decay product of ^225^Ac and decays further to stable ^209^Bi through two branches each involving one α decay and two β^–^ decays.^14,15^^213^Bi can be easily distributed in the form of an ^225^Ac/^213^Bi generator and can be conveniently obtained immediately before application. ^213^Bi-labeled radioligands are used for the treatment of glioma,^16,17^ neuroendocrine tumors,^18^ leukemia,^19,20^ melanoma,^21−23^ and bladder cancer.^24^

A better understanding of tumor biology has led to the development of lipid drug delivery nanosystems with multiple functionalities.^25^ Liposomes have been reported as useful carriers of α emitters.^26−31^ The lipid nanoparticles, e.g., liposomes, cubosomes, and hexosomes, allow multimodal therapy, such as chemotherapy in combination with endoradiotherapy. Larsen et al.^32^ used the commercial drug, doxorubicin encapsulated in pegylated liposomes (Caelyx/Doxil), to perform the synthesis of ^223^Ra-containing radiobioconjugates inside the carriers. Liposome-encapsulated ^223^Ra displayed beneficial physical and radiological characteristics including *in vivo* stability and promising biodistribution properties in mice.

Lipidic liquid-crystalline phases and nanoparticles used in the present work are promising drug carriers since compared to liposomes, they have a much larger interfacial area (400 m^2^/g) and can efficiently bind larger amounts of hydrophilic and hydrophobic drugs.^33,34^ The lipid cubic phase can be characterized as a curved, nonintersecting lipid bilayer with two unconnected water channels.^35^ Monoolein (GMO) is the lipid most commonly used to prepare the cubic phase as it is biodegradable, biocompatible, and nontoxic.^36^ Because of high viscosity, the cubic phase may be difficult to handle; therefore, such a mesophase in the presence of a stabilizer can be dispersed into cubosomes, which are kinetically stable colloidal nanoparticles.^37−39^ Cubosomes retain the internal bicontinuous structure and all of the physicochemical features of the cubic phase that control the drug release kinetics.^40−42^ The versatility of lipidic liquid-crystalline systems in encapsulating and delivering therapeutic agents of different solubilities, charges, and sizes has been proven to overcome problems occurring with other drug carriers, such as small encapsulation efficiency, toxicity, or poor stability.^43−48^ Cubosomes have been explored for anticancer drug delivery^49−51^ and combined delivery of different active agents.^52−55^ We recently reported the enhancement of cytotoxicity achieved when doxorubicin is used together with a complex of ^177^Lu (*t*_1/2_ = 6.7 days) but only after a shorter incubation of HeLa cells.

In the present study, we investigate the liquid-crystalline systems as a platform for combined chemotherapy with α-particle therapy using doxorubicin as a chemotherapeutic agent and a short-lived α-particle emitter ^213^Bi (*t*_1/2_ = 45.6 min).

A chelating ligand, DOTAGA-oleylamine (DOTAGA-OA), was incorporated into cubosomes to form a stable complex with ^213^Bi. The hydrophobic oleylamine tail anchors the ligand in the lipid bilayer of the cubosome structure, while the DOTAGA headgroup with the radioactive dopant is exposed to the water channels. Small-angle X-ray scattering (SAXS) was employed to perform the structural characterization of cubosome formulations, and cryogenic transmission electron microscopy (cryo-TEM) was used for cubosome imaging. The characteristics of the carriers were supplemented with dynamic light scattering (DLS) measurements of their sizes and stabilities. The biological experiments revealed that the presence of the DOTAGA-OA ligand affects the activity of DOX when they are incorporated in the same carrier. This unexpected influence was explained based on the results of release studies showing the pH-dependent molecular interaction between the two moieties. Taking these interactions into account allowed for the elaboration of the optimal way for the drugs, doxorubicin and complexed ^213^Bi delivery, to achieve the increased cytotoxic effect and obtain more effective action toward the malignant cells.

## 2 Experimental Section

### 2.1 Reagents

Monoolein (1-oleoyl-rac-glycerol, GMO) of purity ≥ 99%, doxorubicin hydrochloride (DOX), and Pluronic F-127 used for cubosome preparation were all obtained from Sigma-Aldrich (St. Louis, MO). To prepare MES buffer, 0.1 M 2-(*N*-morpholino) ethanesulfonic acid or 0.1 M 2-amino-2-(hydroxymethyl)-1,3-propanediol was titrated with 0.1 M NaOH or 0.1 M HCl (Polish Chemicals Co., Gliwice, Poland) to obtain the required pH of the buffer. Milli-Q water (18.2 MΩ cm^–1^; Millipore, Bedford, MA) was used to prepare all solutions.

DOTAGA-OA ligand was synthesized according to a previously described procedure.^55^ Analysis glass microfiber sheets (Agilent, Santa Clara, CA) were used for instant thin-layer chromatography (ITLC).

Cell experiments were performed with the use of the following materials: RPMI-1640 medium, fetal bovine serum, phosphate-buffered saline (PBS), trypsin–EDTA, and penicillin/streptomycin solutions from Biological Industries (Biological Industries, Beth Haemek, Israel); dimethyl sulfoxide (DMSO) (Sigma-Aldrich, St. Louis, MO); CellTiter-96 aqueous one solution reagent (MTS compound) from promega (Promega, Madison, WI); FITC Annexin V and propidium iodine (PI) staining solution from BD Biosciences (BD Biosciences, San Jose, CA); and Hoechst 33258 from Thermo Fisher Scientific, Inchinnan, Scotland.

A human-derived HeLa cancer cell line was obtained from the American Type Culture Collection (ATCC, Rockville, MD) and was cultured in RPMI-1640 medium supplemented with 10% fetal bovine serum and 1% penicillin/streptomycin. The cells were grown at 37 °C in a humidified atmosphere containing 5% CO_2_.

### 2.2 Radionuclides

^213^Bi was obtained from the radionuclide generator loaded with its longer-lived mother nuclide ^225^Ac. ^225^Ac/^213^Bi standard generator with 0.3 mL of AG MP-50 resin in perfluoroalkoxy tubing with polypropylene fittings equipped with silicone tubing was used (Joint Research Centre, European Commission, Karlsruhe, Germany). The resin was preconditioned using 6 M HCl, 0.05 M HNO_3_, 6 M HNO_3_, and finally, 0.05 HNO_3_. Directly before loading ^225^Ac, the resin was washed with 2 mL of 4 M HNO_3_. A 600 μL solution of 0.1 M HCl/0.1 M NaI was used as an eluent. The strong affinity of Bi(III) for complexation with iodide was used for the selective elution of ^213^Bi from the cation-exchange resin as anionic BiI_4_^–^/BiI_5_^2–^ species.^56^ Breakthrough of ^225^Ac after this procedure was lower than 5%. The generator was ready for elution 3 h after loading, with an efficiency of 93.8%.

### 2.3 Preparation of Cubic Phases and Cubosomes

To prepare the bulk nondoped cubic phase, an appropriate amount of molten monoolein (GMO) and MES buffer solution was mixed together. The ratio of components was chosen on the basis of the phase diagrams for the GMO/water system.^57^ Cubic phases with incorporated DOX were prepared similarly as described above, but first, DOX was dissolved in the buffer solution and then mixed with the appropriate amount of molten GMO. DOTAGA-OA was dissolved first in a chloroform/methanol mixture (4:1 v/v) and then used for the preparation of mesophases. Next, solvent evaporation was performed and DOTAGA-OA was mixed with molten GMO. Then, MES buffer solution was added. The final compositions of cubic phases with DOTAGA-OA are presented in the Supporting Information, Table S1. To prepare mesophases loaded with DOX and DOTAGA-OA together, a chloroform/methanol mixture (4:1 v/v) was used as a solvent to dissolve DOTGA-OA. Solvent evaporation was performed, and DOTAGA-OA was then mixed with molten GMO. Next, DOX in MES buffer solution was added. Samples were stabilized for at least 24 h to obtain transparent, viscous, and homogeneous cubic phases. The obtained samples were stored in sealed vials at room temperature and protected from light.

Cubosomes were synthesized according to a previously described protocol.^55^ A top-down approach was employed. Briefly, cubosome preparation involved the hydration of GMO with MES buffer solution and Pluronic F-127 stabilizer. Next, the samples were sonicated in a Sonic 0.5 ultrasonic bath (Polsonic, Poland). On the basis of radiolabeling conditions, the amount of DOTAGA-OA in the cubosomal formulations was selected.

In Table 1, the final composition of the cubic phases and cubosomes is presented.

**Table 1:** Cubic Phases and Cubosome Final Compositions

cubic phase	final compositions of samples	ratio of the components (wt %)
blank	buffer/GMO	40/60
DOX	buffer/GMO/DOX	39.8/60/0.2
DOX DOTAGA-OA	buffer/GMO/DOX/DOTAGA-OA	39.8/58.1/0.2/1.9
cubosomes		
blank	buffer/GMO/F-127	94.62/4.84/0.54
DOX	buffer/GMO/DOX/F-127	94.58/4.86/0.02/0.54
DOTAGA-OA	buffer/GMO/DOTAGA-OA/F-127	94.45/4.85/0.16/0.54
DOX DOTAGA-OA	buffer/GMO/DOX/DOTAGA-OA/F-127	94.44/4.84/0.02/0.16/0.54

### 2.4 Characterization Methods of Mesophases

#### 2.4.1 Small-Angle X-ray Scattering (SAXS)

SAXS was employed to determine the structure of cubic phases and cubosomes. SAXS measurements were carried out on a Bruker Nanostar system equipped with a Vantec 2000 area detector (Madison, WI) and Cu Kα radiation. X-ray measurements allowed us to determine the properties of liquid crystals such as the crystal lattice parameter and water channel diameter (Supporting Information, 1S).

#### 2.4.2 Dynamic Light Scattering (DLS)

DLS (Zetasizer Nano ZS Malvern, U.K.) was used to determine the average size, polydispersity (PDI), and zeta potential (ζ) of cubosomes. The measurements were performed at 25 °C, and the viscosity of pure water was assumed. Results are presented as an average of three separate measurements.

#### 2.4.3 Cryogenic Transmission Electron Microscopy (cryo-TEM)

Three microliters of cubosome dispersions were applied onto glow-discharged Quantifoil R2/2 holey carbon grids and plunge-frozen in liquid ethane using a Vitrobot Mark IV device (Thermo Fisher Scientific, Waltham, MA). Two-dimensional electron cryomicroscopy images were taken in the linear mode on a Thermo Fisher Glacios TEM (Waltham, MA) microscope operating at 200 kV, equipped with a 4k × 4k Falcon 3EC direct electron detection camera and EPU 2.10 software. The following parameters were used for collecting images: magnification of 92k corresponding to a pixel size of 0.15 nm (1.5 Å) at the specimen level; defocus set to 4.0 and 3.5 μm; and the total electron dose of approximately 40 e/Å^2^.

#### 2.4.4 Electrochemistry

Electrochemical experiments were performed with a CHI 700B bipotentiostat (CH Instruments Inc., Austin, TX) that has a standard three-electrode arrangement in buffered solution. Ag/AgCl was used as the reference electrode and a platinum foil was used as the counter electrode. The working electrode was a glassy carbon electrode (GCE, *A* = 0.07 cm^2^) modified with the cubic phase. Before the experiments, the working electrode was polished on the alumina of decreasing size (from 0.3 to 0.05 μm) with a polishing cloth. The electrodes were subsequently sonicated to remove adhered alumina particles, rinsed with ethanol and water, and left to dry. Cyclic voltammetry (CV) and differential pulse voltammetry (DPV) were used to determine the doxorubicin release from the mesophase. The mesophase was deposited on the electrode surface by means of a Teflon cap, which kept the thickness of the mesophase layer equal to 0.5 mm and the volume of the layer remained constant in all experiments. After covering the electrode with the cubic phase layer, it was immediately immersed in the deoxygenated buffer solution. All experiments were carried out at room temperature in solutions deoxygenated by purging with argon (99.999%) for 15 min, and then an argon blanket was kept over the solution surface. For each type of measurement, triplicate experiments were performed.

#### 2.4.5 Release Studies

To obtain the release profile from cubosomes, nanoparticles with DOX or DOX and DOTAGA-OA together were placed in the dialysis membrane (MWCO 12–14 kDa) and submerged in 50 mL of MES buffer, pH 5.5. To determine the release rate of DOX from nanoparticles, UV–vis spectroscopy was applied using a UV–vis Cary 60 spectrophotometer (Agilent Technologies, Warsaw, Poland). At least 10 dilutions in the concentration range of 0.0005–0.1 mg/mL were used to obtain the standard calibration curve for DOX solution in 0.1 M MES buffer at pH 5.5. The wavelength range of 600–250 nm with the characteristic λ at 480 nm was selected to measure the absorption spectra of the DOX buffer solutions. All of the measurements were performed at room temperature (25 °C).

### 2.5 Protocol for Cubosome Radiolabeling

Six hundred microliters of 0.1 M HCl/0.1 M NaI elution from ^213^Bi generator (20 MBq) was added to 6 μL of cubosomes doped with DOTAGA-OA (10 nmol in 6 μL of cubosome dispersion). Samples were placed in Eppendorf tubes, and 400 μL of tris buffer (0.1 M, pH 7.0) was added. The solution was heated at 95 °C for 20 min and cooled to room temperature. The instant thin-layer chromatography (ITLC) method with citrate buffer (0.5 M, pH 5.5) as the mobile phase and glass microfiber TLC plates was used to determine the labeling efficiency. In this method, the unbound ^213^Bi moves with the front of the solvent (*R*_f_ = 1) and labeled conjugates remain at the baseline (*R*_f_ = 0). The radioactivity distribution on the ITLC strips was measured by a Cyclone Plus Phosphor Imager (Perkin–Elmer life and analytical sciences, Shelton, CT) and analyzed using Optiquant software supplied by the manufacturer.

### 2.6 *In Vitro* Cytotoxicity Assay: MTS

To determine the cell metabolic activity, the MTS test was used. Cytotoxicity studies were carried out for cubosome dispersions containing ^213^Bi-DOTAGA-OA and ^213^Bi-DOTAGA-OA with DOX. Blank cubosomes (GMO concentration: 55 μg/mL) and cubosomes doped with DOX (DOX concentration: 0.2 μg/mL) were used as controls.

HeLa cells were plated in 96-well plates at a density of 2 × 10^3^ cells per well at 37 °C in a humidified environment of 5% CO_2_ for 24 h. Then, cells were washed with PBS and increasing doses of ^213^Bi (500, 1000, and 2000 kBq/mL) in cubosomes suspended in the cell culture medium solution were added at a volume of 100 μL per well. After 24 h, the solution was removed, and cells were washed with PBS and treated with blank cubosomes or cubosomes doped with DOX suspended in cell culture medium. Further, the treated cells were incubated for the next 24, 48, and 72 h. The MTS assays were performed using the CellTiter-96 AQueous-Non-Radioactive Cell Proliferation Assay (Promega, Mannheim, Germany). The absorbance of the formazan product was measured at 490 nm using a microplate reader (Berthold, Bad Wildbad, Germany). The results are expressed as the percentage of cell viability relative to the mean of the control groups (cells grown in medium only).

### 2.7 Flow Cytometry Assays

Two flow cytometry tests were performed—the apoptosis and cell cycle assays. For this purpose, HeLa cells cultured in RPMI-1640 medium with 10% FBS and 1% penicillin–streptomycin supplementation were used. HeLa cells were seeded (4 × 10^5^ per well) in six-well plates and incubated for 24 h at 37 °C in the 5% CO_2_ atmosphere. Next, the compounds presented in Table 2 were added to the wells and incubated for 24 and 48 h. Cells for apoptosis testing were trypsinized, washed twice with cold phosphate buffer (PBS), and resuspended in Annexin V binding buffer. Then, 5 μL of FITC-labeled Annexin V and 5 μL of propidium iodide (PI) were added followed by incubation for 15 min at 37 °C in the dark.

**Table 2:** Procedures of Dosing of Samples and Cell Culture Media

short name of sample delivered sequentially or together	1st dose (24 h)	2nd dose (48 h)
control	cell culture medium	cell culture medium
^213^Bi-Cub/Cubo	^213^Bi-DOTAGA-OA in cubosomes	blank cubosomes
Cubo/DOX-Cubo	blank cubosomes	cubosomes doped with 0.2 μg/mL DOX
^213^Bi-Cubo/DOX-Cubo	^213^Bi-DOTAGA-OA in cubosomes	cubosomes doped with 0.2 μg/mL DOX
(^213^Bi-Cubo + DOX)-Cubo	drugs added together in one cubosome	

Samples for the cell cycle analysis were prepared identically as samples for apoptosis assay. After washing twice with cold PBS, cells were suspended in 70% cold EtOH and then frozen for 90 min. After centrifugation, the cells were washed twice with PBS, and 20 μL of propidium iodide (PI) and 2 μL of RNase were added. Apoptosis and cell cycle assays were performed with the use of flow cytometry FACSCelesta (BD Biosciences, San Jose, CA), while the analysis of the results was carried out using FACSDiva software v8.0 (BD Biosciences, San Jose, CA).

### 2.8 Spheroids

Spheroid formation was initiated by seeding HeLa cells into a 96-well plate with the ultralow attachment surface (Corning, NY). They were grown to the size of 375 μm. Next, three-dimensional (3D) aggregates of cells were treated with 500 and 2000 kBq/mL of ^213^Bi in cubosome solutions. Spheroids were incubated with the radiocompounds for 24 h, and then the solution was removed. Spheroids were treated with blank cubosomes or cubosomes doped with DOX suspended in cell culture medium. After 24 h incubation, spheroids were suspended in fresh medium, which was then replaced every 2 days. Additionally, spheroids treated with an activity concentration of 0.5 MBq/mL were also stained with fluorescent reagents such as propidium iodide and Hoechst 33258. The growth of individual 3D cell culture models was measured for up to 12 days after treatment. To determine the diameter of spheroids, a Primovert microscope with an Axiocam 305 color (Zeiss, Jena, Germany) was applied. Measurements were performed with ZEN 3.0 lite software (Zeiss, Jena, Germany).

### 2.9 Statistical Analysis

GraphPad Prism version 8.0 software (GraphPad Software Inc., San Diego, CA) was used to analyze the experimental data. To determine the cytotoxicity (MTS assay, flow cytometry analysis), values between groups were compared using one-way ANOVA. The results are presented as the mean ± standard error of the mean (SEM) and were considered statistically significant when *p* ≤ 0.05, *p* ≤ 0.01, *p* ≤ 0.001, and *p* ≤ 0.0001.

## 3 Results and Discussion

### 3.1 Characterization of Cubic Phases and Cubosomes

#### 3.1.1 Small-Angle X-ray Scattering (SAXS)

**Figure 1:**
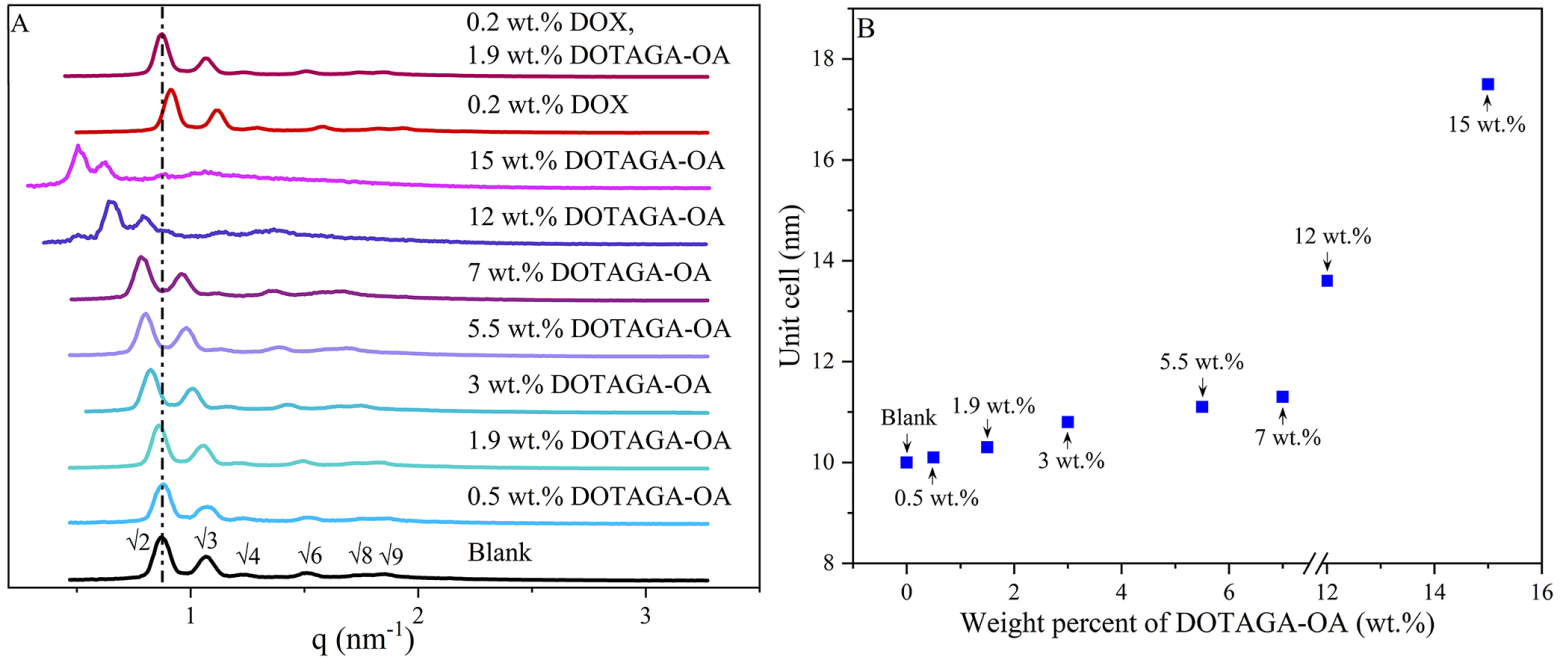
(A) SAXS profile of the different cubic phases at 25 °C. (B) Unit cell dependence of weight percent of DOTAGA-OA or DOX and DOX together with DOTAGA-OA in cubic phases.

SAXS was employed to characterize the structure of the various lipidic liquid-crystalline phases. Figure 1A shows the SAXS diffraction patterns obtained for mesophases doped with different amounts of DOTAGA-OA, DOX or DOX, and DOTAGA-OA together.

For the blank cubic phase, the diffraction patterns showed reflections in the ratios of √2, √3, √4, √6, √8, and √9, which corresponds to the double diamond cubic (*Pn*3*m*) symmetry and a lattice parameter of 10.0 nm. Structural parameters are presented in Table 3. The progressive incorporation of DOTAGA-OA up to 15 wt % did not affect the symmetry of the phase but instead resulted in an increased unit cell parameter (17.5 nm) and water channel dimensions of up to 10.1 nm (Figure 1B). This occurs as a result of electrostatic repulsions of the negatively charged DOTAGA headgroup exposed to the water channel of the cubic phase. Mesophase with an addition of both DOX (0.2 wt %) and DOTAGA-OA (1.9 wt %) exhibited a minor increase of the lattice parameter (10.1 nm). Therefore, the dopants did not affect the symmetry of the phase.

**Table 3:** Properties of Cubic Phases Determined Using SAXS

phase	unit cell (nm)	diameter of the aqueous channel (nm)
blank	10.0	4.2
0.5 wt % DOTAGA-OA	10.1	4.3
1.9 wt % DOTAGA-OA	10.3	4.5
3 wt % DOTAGA-OA	10.8	4.9
5.5 wt % DOTAGA-OA	11.1	5.1
7 wt % DOTAGA-OA	11.3	5.2
12 wt % DOTAGA-OA	13.6	7.0
15 wt % DOTAGA-OA	17.5	10.1
0.2 wt % DOX	9.8	4.1
0.2 wt % DOX, 1.9 wt % DOTAGA-OA	10.1	4.3

**Figure 2:**
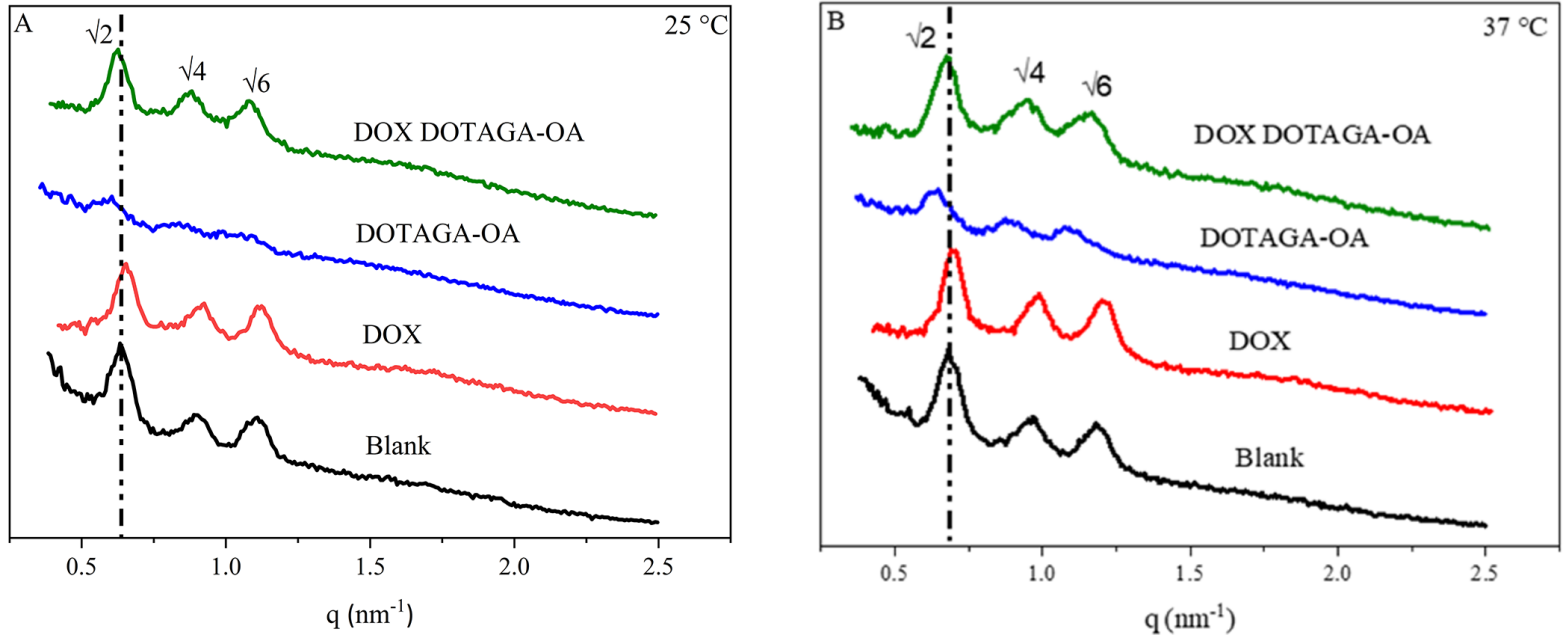
1D diffraction patterns of cubosome formulations at (A) 25 °C and (B) 37 °C.

SAXS was also used to elucidate the type and structural parameters of the cubosomes doped with DOX, DOTAGA-OA, DOX, and DOTAGA-OA together. Figure 2 presents one-dimensional diffraction patterns for obtained systems showing reflections in the ratio of √2, √4, and √6, which corresponds to the *Im*3*m* (primitive) structure.^58,59^

The addition of a small amount of DOX (0.02 wt %) did not significantly alter the properties of the cubosomes (Table 4). Incorporation of DOTAGA-OA (0.16 wt %) into cubosomes resulted in an increase in the crystallographic unit cell parameter, but the primitive cubic structure was preserved. Furthermore, the introduction of the DOX and DOTAGA-OA together did not alter the internal structure of the cubosomes. The lattice parameter is decreased for all samples at 37 °C.

**Table 4:** Characterization of Cubosome Formulations Using SAXS and DLS

cubosome formulation	unit cell (nm)	diameter of the aqueous channel (nm)	hydrodynamic diameter (nm)	PDI	ζ potential (mV)
blank	14.0^a^	5.0^a^	140 ± 5	0.18 ± 0.02	–29 ± 0.9
	12.9^b^	4.3^b^			
DOX	13.7^a^	4.8^a^	160 ± 10	0.19 ± 0.01	–24 ± 0.4
	12.7^b^	4.2^b^			
DOTAGA-OA	14.7^a^	5.4^a^	130 ± 15	0.12 ± 0.02	–20 ± 0.6
	14.0^b^	5.0^b^			
DOX DOTAGA-OA	14.2^a^	5.1^a^	150 ± 12	0.13 ± 0.03	–17 ± 0.8
	13.0^b^	4.4^b^			

a 25 °C.

b 37 °C.

#### 3.1.2 Physicochemical Characterization of Cubosomes

Dynamic light scattering (DLS) was employed to provide information on the physicochemical properties of cubosomal formulations. The hydrodynamic diameter, polydispersity index (PDI), and zeta potential are presented in Table 4. The mean diameter of nonloaded cubosomes was 140 ± 5 nm, and the PDI value was close to 0.18. For the DOTAGA-OA-doped cubosomes, the particle diameter was slightly lower (130 ± 15 nm) with a PDI value of approximately 0.12. For cubosomes loaded with DOX or DOX and DOTAGA-OA together, the diameter was close to 160 ± 10 nm with a PDI of 0.19 and 150 ± 12 with a PDI of 0.13, respectively. The obtained PDI values of cubosomes indicated homogeneity of the formulations and also demonstrated that the structures do not aggregate. The ζ potentials obtained for the cubosomes were close to −29 mV for nondoped cubosomes and −24 mV for DOX-loaded nanoparticles. For cubosomes with DOTAGA-OA or DOX and DOTAGA-OA together, zeta potentials were −20 and −17 mV. These values implicated the stability of the obtained nanoparticles.

**Figure 3:**
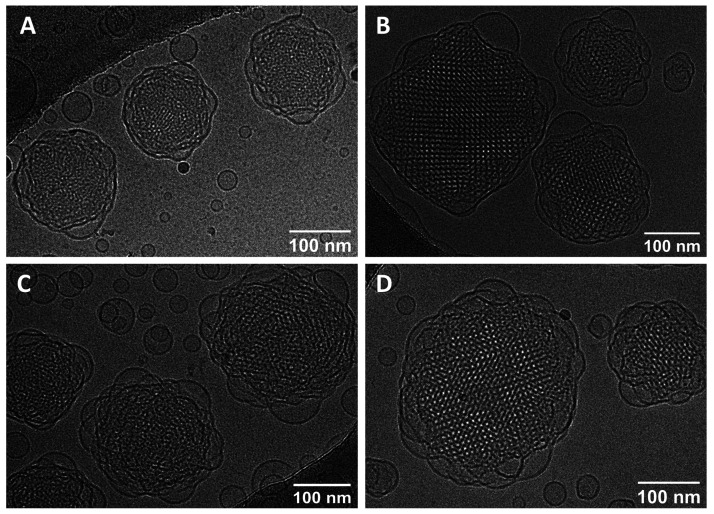
cryo-TEM images of (A) blank cubosomes, (B) cubosomes loaded with DOX, (C) DOTAGA-OA, and (D) DOX and DOTAGA-OA.

The cryo-TEM images obtained for blank and doped cubosomes showed the well-ordered internal structure of the nanoparticles (Figure 3). Cryo-TEM images showed that cubosomal dispersions also contain some fraction of vesicles in agreement with other reports.^60,61^

### 3.2 Release Studies of Doxorubicin in the Absence and Presence of DOTAGA-OA in the Cubic Phase Monitored by Electrochemical Methods

The electrochemical behavior of DOX incorporated in cubic phases was monitored using cyclic (CV) and differential pulse voltammetry (DPV) at pH 5.5, which corresponds to the tumor microenvironment. DOX is an electroactive molecule with one quinone (Q) and one hydroquinone (QH2) groups. The quinone or hydroquinone moieties undergo 2e^–^/2H^+^ reduction and oxidation processes, respectively (SI, Scheme 1S). The redox properties of adriamycines were described by Komorsky-Lovrić,^62^ and the redox properties of DOX in cubic phases were described in more detail in our previous work.^45^

**Figure 4:**
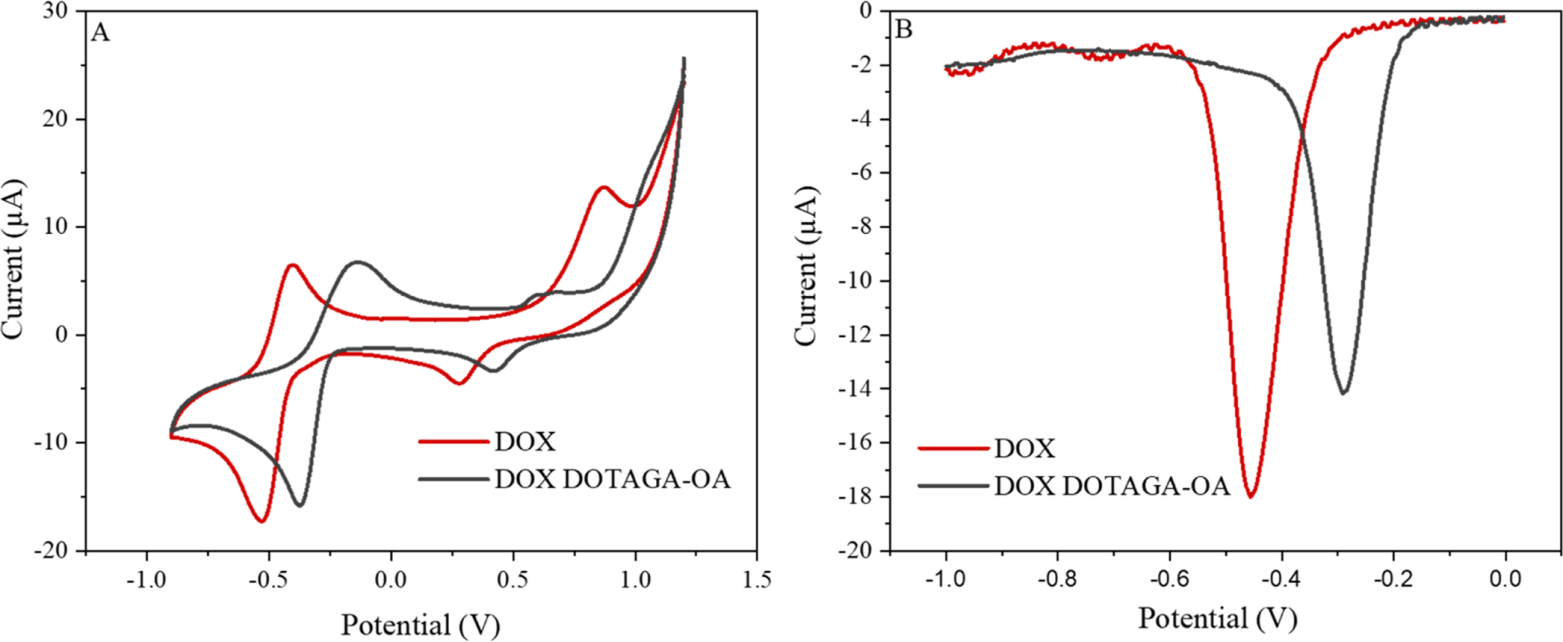
(A) Cyclic voltammograms for DOX incorporated into mesophase with or without the DOTAGA-OA dopant. Scan rate: 100 mV/s, pH 5.5. (B) DPV recorded on GCE modified with DOX-containing cubic phases in 0.1 M MES buffer at pH 5.5. Amplitude: Δ*E* = 50 mV, pulse time: *t*_p_ = 50 ms.

In the cubic phase, DOX undergoes reduction at approximately −0.5 V corresponding to the reduction of the 5,12-diquinone groups, while less reversible oxidation of the hydroquinone unit occurs at +0.74 V (Figure 4A). The reduction peak is used for determining the release kinetics of the drug from the cubic phase. Interestingly, when the cubic phase is doped with both DOX and DOTAGA-OA, the reduction peak of DOX is shifted toward more positive potential values. The change in the peak position indicates that the electron acceptor properties of the molecule become stronger either due to lower pH in the immediate environment of the reducing species (lower pH value due to the presence of the carboxylic moieties in the DOTAGA headgroup) or due to interactions between the DOX and DOTAGA-OA stronger for the reduced form of the drug.

This shift of reduction peak potentials is clearly seen in the voltammograms recorded by DPV (Figure 4B). Additionally, the height of the peak is decreasing, indicating that the diffusion of DOX to the electrode surface is slower when DOX is involved in the interactions with the macrocyclic ligand DOTAGA-OA. DOX at pH 5.5 is positively charged, while the DOTAGA-OA ligand is in its deprotonated form, which facilitates the interaction of the drug with the ligand. This effect will have to be taken into account in the simultaneous delivery of both drugs: chemo- and radiotherapeutic in the combined therapy.

**Figure 5:**
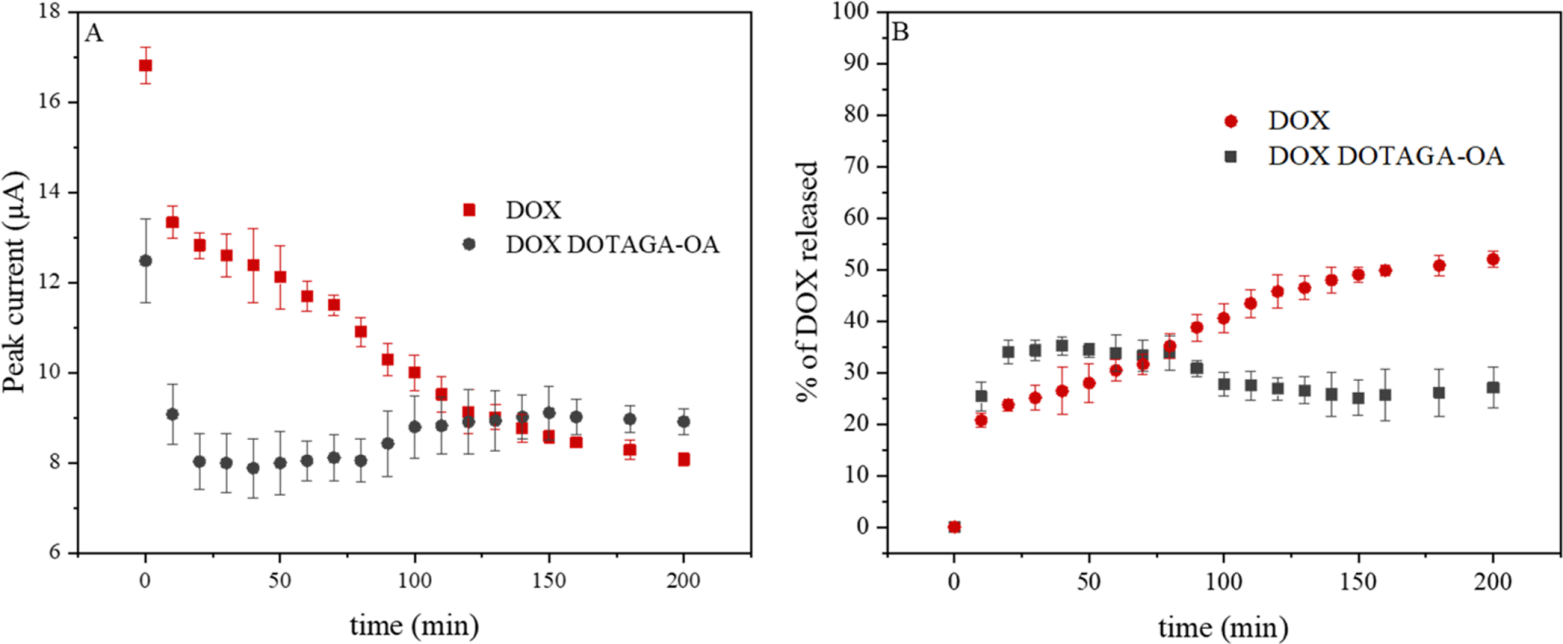
(A) Release profile of DOX-containing or DOX- and DOTAGA-OA-containing mesophases at pH 5.5. (B) The current normalized release profile of DOX from the mesophases plotted as a percentage of DOX released *vs* time.

The release study of DOX was carried out to determine how the presence of DOTAGA-OA ligand in the cubic phase influences the release profile of the incorporated chemotherapeutic agent. The release profiles of DOX from mesophases were determined based on the changes of peak currents recorded by DPV with time following immersion of the electrodes into the buffer solution. With time, the peak currents decreased, reflecting the elution of the drug from the phase (Figures 5 and 1S (SI)). To quantify the release and to determine the release kinetics of the peak, current values were normalized to *I*/*I*_0_, where *I*_0_ is the DOX peak current at *t*_0_ (Figure 5B).

**Figure 6:**
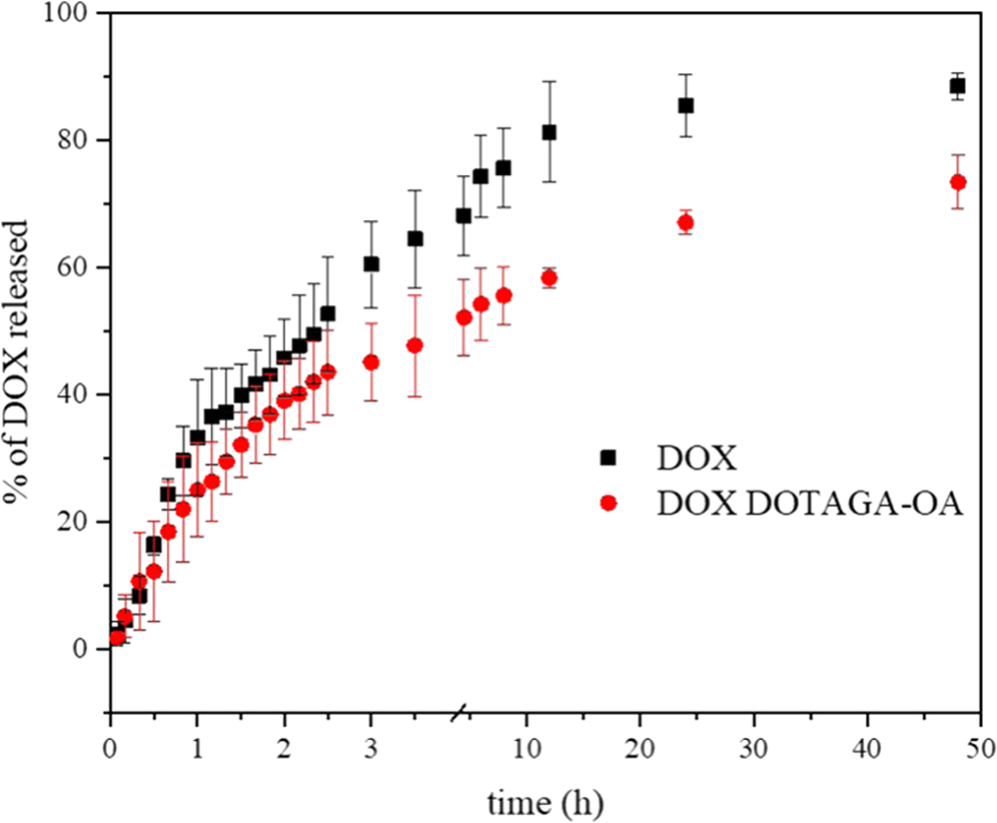
Release profile of DOX-containing or DOX- and DOTAGA-OA-containing cubosomes measured by UV–vis spectroscopy.

Comparison of DOX release profiles reveals that the release of DOX in a measured time interval is retarded when the mesophase is doped with DOTAGA-OA. Similar effect was observed in the case of cubosomes (Figure 6). Release studies performed in a longer time interval −48 h of measurement confirmed that the release of DOX was slower in the presence of DOTAGA-OA ligand.

In the acidified medium (pH = 5.5) typical for cancer cell environment, the positively charged DOX is placed predominantly in the aqueous channels of the cubic phase, as previously described,^63^ and therefore, it diffuses fast from the cubic phase. However, interaction with DOTAGA-OA neutralizes its charge, which allows its penetration into the lipidic bilayers surrounding the water channels of the cubic phase. Diffusion from the lipidic part of the cubic phase is much slower. This explains why the presence of DOTAGA-OA ligand and its interaction with DOX delays the release of DOX from the cubic mesophase.

### 3.3 *In Vitro* Cytotoxicity Results of the MTS Assay

**Figure 7:**
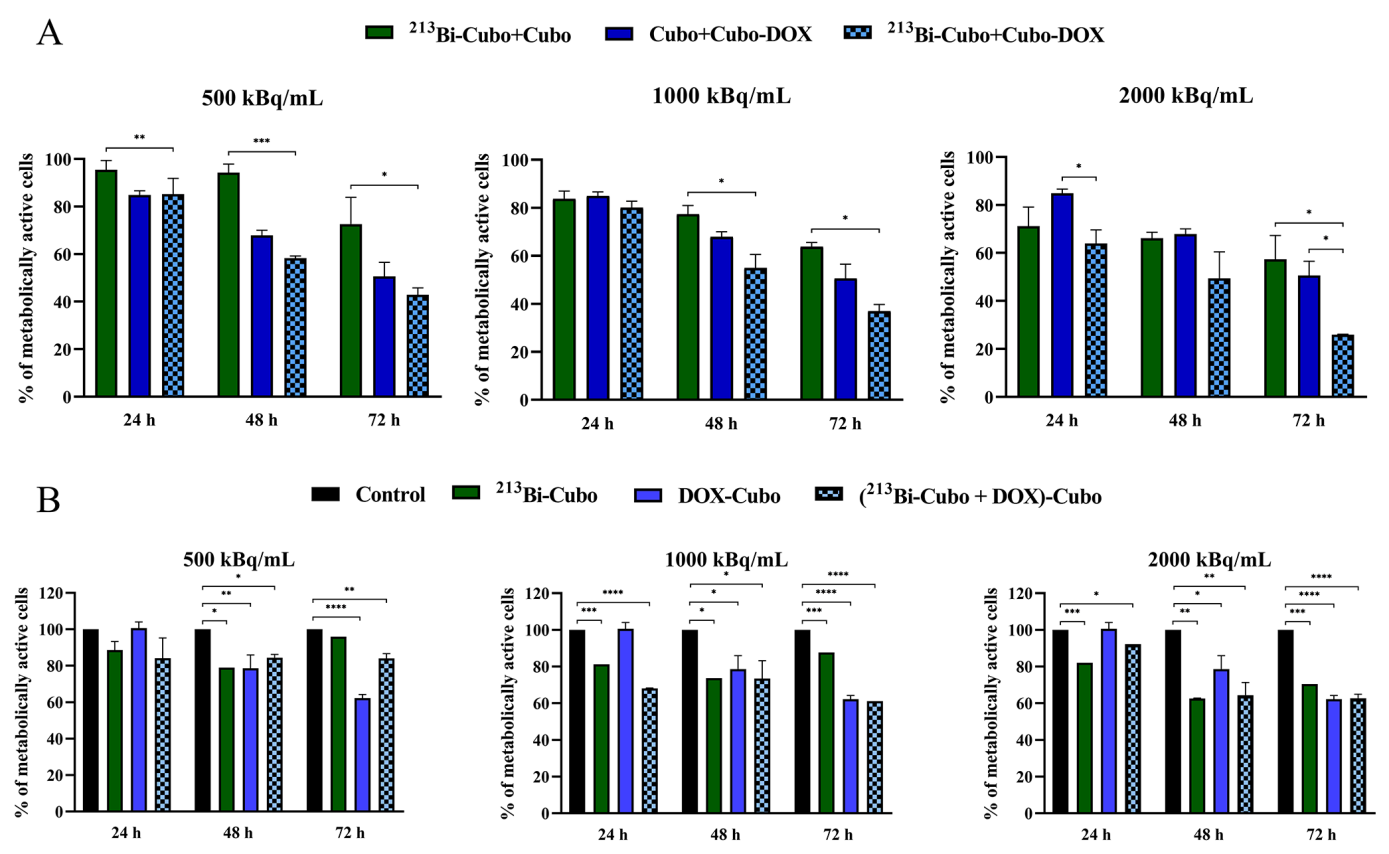
(A) Viability of HeLa cells treated sequentially with ^213^Bi-Cubo/Cubo, Cubo/DOX-Cubo, and ^213^Bi-Cubo/DOX-Cubo after 24, 48, and 72 h of incubation, respectively. ^213^Bi-Cubo/Cubo and Cubo/DOX-Cubo were used as a control. (B) Viability of HeLa cells treated with ^213^Bi-Cubo, DOX-Cubo, and (^213^Bi-Cubo + DOX)-Cubo after 24, 48, and 72 h of incubation, respectively. As a control, nontreated cells were used. Data points and SD are from at least three measurements. Statistical significance was considered if *p* ≤ 0.05 (*), *p* ≤ 0.01 (**), *p* ≤ 0.001 (***), and *p* ≤ 0.0001 (****).

After the labeling of cubosomes with ^213^Bi (radiolabeling yield > 98%) and incorporation of chemotherapeutic, the obtained radioconjugates were tested *in vitro*. Cytotoxicity of cubosome dispersions containing DOTAGA-OA-complexed ^213^Bi and DOX, at three measurement points (24, 48, and 72 h) including three various doses (500, 1000, and 2000 kBq/mL) and a DOX concentration of 0.2 μg/mL (Figure 7A), was performed using the MTS assay. As preliminary MTS studies where DOX and ^213^Bi were incorporated simultaneously, cubosomes showed less toxicity (Figure 7B) of radioconjugate compared to cubosomes doped only with DOX due to the interactions of DOTAGA-OA ligand with DOX, as discussed above. The next experiments were performed using cubosomes containing the following components separately: complexed ^213^Bi (^213^Bi-Cubo) and DOX-Cubo added to the cells sequentially.

The obtained viability results (Figure 7A) showed that the tested compounds caused a decrease in the metabolic activity of HeLa cells in a time- and dose-dependent manner. The addition of DOX-Cubo significantly reduces the viability of HeLa cells, and its impact on toxicity is dominant in comparison to toxicity deriving from radiation but only at lower doses −500 and 1000 kBq/mL. The concentration of 0.2 μg/mL of DOX and 2000 kBq/mL of ^213^Bi loading cubosomes induce similar cytotoxicity in HeLa cells. In the case of ^213^Bi-Cubo/DOX-Cubo, the cytotoxicity effect is higher than for cubosomes doped only with ^213^Bi radionuclide or chemotherapeutic. The viability of HeLa cells exposed to ^213^Bi-Cubo and DOX-Cubo is the lowest (26%) with the use of 2000 kBq/mL of radioconjugate after 72 h of incubation. The results clearly demonstrated that the combined therapy with the highest dose of ^213^Bi and 0.2 μg/mL of DOX encapsulated separately in cubosomes seems to be the best option for the treatment of cancer cells.

### 3.4 Apoptosis and Cell Cycle Studies

To determine whether the inhibition effect of DOX and α radiation incorporated in cubosomes is associated with triggering the programmed cell death pathways, apoptosis in human HeLa cancer cells was analyzed. The programmed cell death was evaluated by flow cytometry using Annexin V-FITC and PI fluorescence staining assay (Figure 2S).

**Figure 8:**
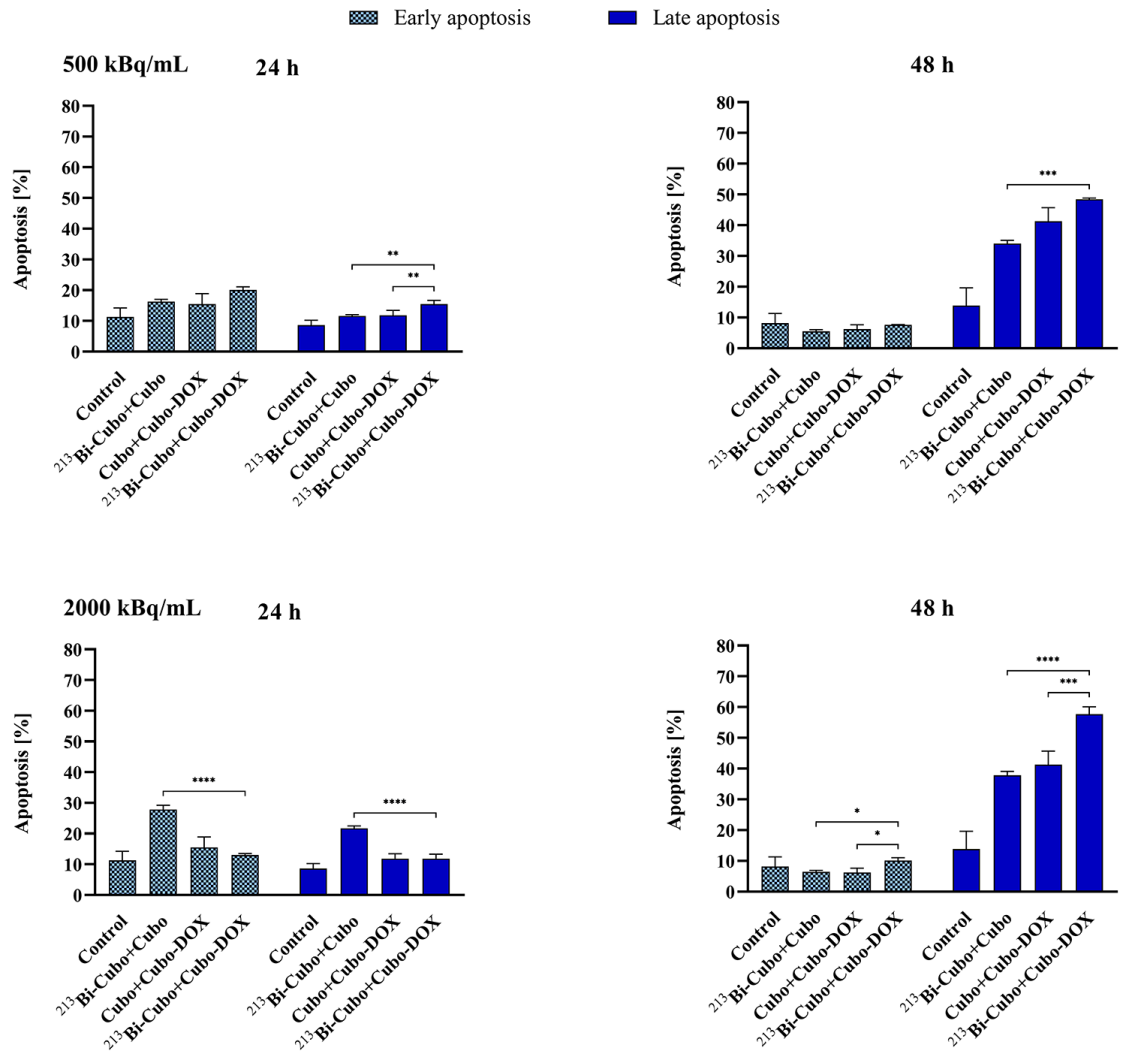
Graphs showing the percentage of apoptotic cells *in vitro* treated sequentially with ^213^Bi-Cubo/Cubo, Cubo/DOX-Cubo, and ^213^Bi-Cubo/DOX-Cubo at a DOX concentration of 0.2 μg/mL and 500 and 2000 kBq/mL of ^213^Bi radionuclide after 24 and 48 h of incubation. As a control, ^213^Bi-Cubo/Cubo and Cubo/Cubo-DOX were used. Data points and SD are from at least three measurements. Statistical significance was considered if *p* ≤ 0.05 (*), *p* ≤ 0.01 (**), *p* ≤ 0.001 (***), and *p* ≤ 0.0001 (****).

The fraction of early apoptotic cells did not increase significantly upon the treatment with cubosomes doped with ^213^Bi and DOX compared to the control. Moreover, fewer early apoptotic cells were detected after 48 h than 24 h. Late apoptosis in HeLa cells appears to be more pronounced at higher doses, and an increase in induction is seen mostly in a time-dependent manner, which is in agreement with studies performed for another α emitter, ^225^Ac radionuclide.^58^ The highest apoptosis (57.7%) was observed in cells treated sequentially with 2000 kBq/mL ^213^Bi-Cubo/DOX-Cubo after 48 h of incubation, whereas ^213^Bi-Cubo/Cubo and Cubo/DOX-Cubo induced lower apoptosis, 37.8 and 41.3%, respectively (Figure 8).

The combined ^213^Bi-Cubo/DOX-Cubo treatment resulted in the enhanced induction of apoptosis as compared to single treatments with either ^213^Bi-Cubo or DOX-Cubo. Overall, these findings indicate that the cell death of cubosomes encapsulated DOX and ^213^Bi occurs mainly through apoptosis.

**Figure 9:**
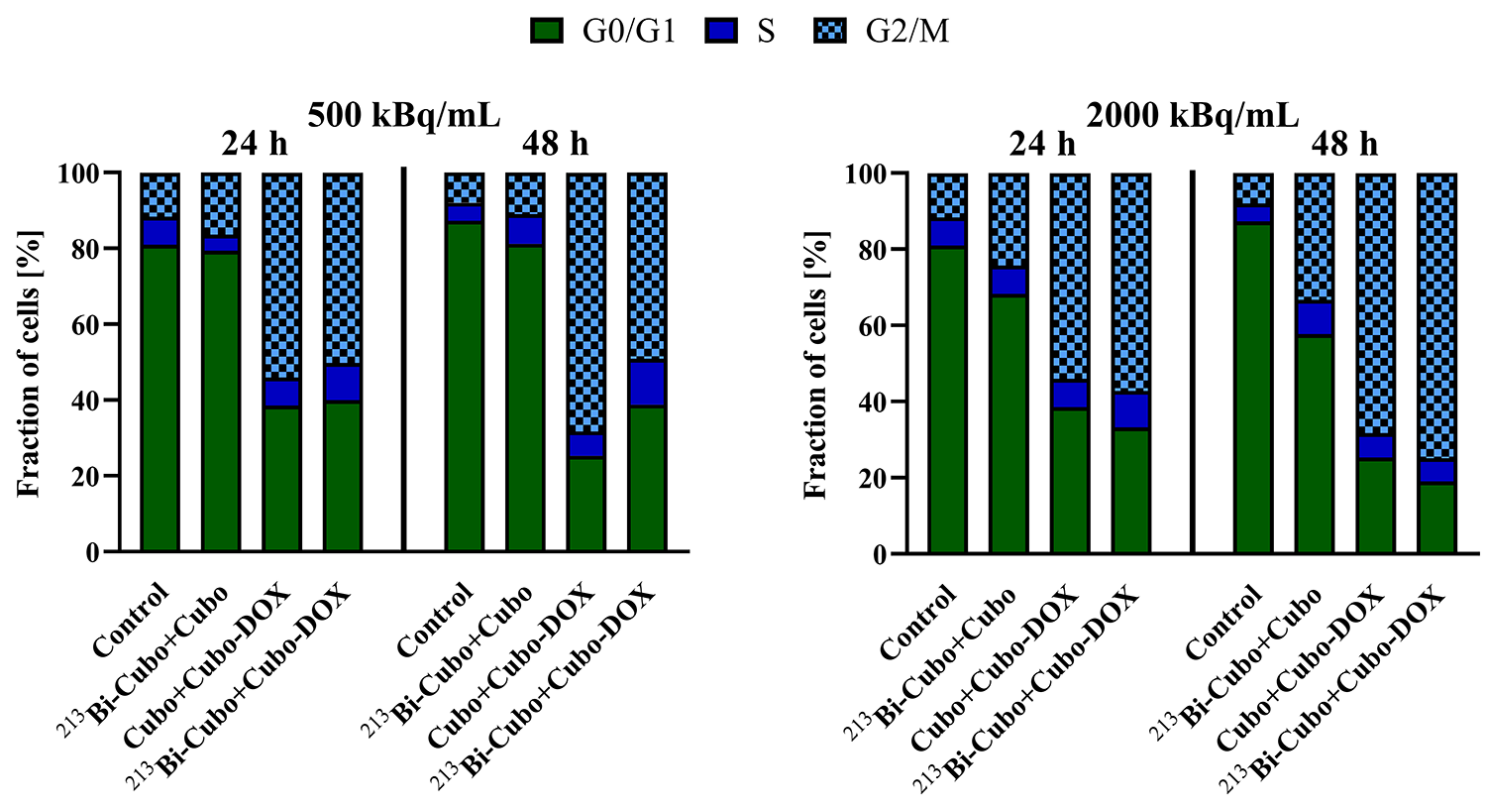
Cell cycle arrest in HeLa cells induced by ^213^Bi-Cubo/Cubo, Cubo/DOX-Cubo, and ^213^Bi-Cubo/DOX-Cubo. Percentages of cells in G0/G1, S, and G2/M phases (*n* = 3 ± SD).

To further characterize the effects of doped cubosomes on HeLa cells, the cell cycle was analyzed (Figure 3S). HeLa cell line was exposed to α-radiation with radioactivity levels of 500 and 2000 kBq/mL and to doxorubicin at a concentration of 0.2 μg/mL, separately incorporated in cubosomes. As shown in Figure 9, Cubo/DOX-Cubo and ^213^Bi-Cubo/DOX-Cubo treatments significantly increased the percentage of cells arrested at the G2/M phase after 24 and 48 h of incubation and reduced the number of cells in G0/G1, compared to the untreated control. The influence of α radiation (^213^Bi-Cubo/Cubo) on the percentage of cells in the G2/M phase was not as high as for chemotherapeutic (Cubo/DOX-Cubo) but increased with time and used dose. A maximum increase in the G2/M arrest (75%) was observed when cells were treated with 2000 kBq/mL of ^213^Bi-Cubo/DOX-Cubo after 48 h of incubation (Figures 3S and 9). However, at a 500 kBq/mL dose of radioconjugate, the cell arrest was lower. Overall, these results are in agreement with literature data where α-radiation^64−66^ and doxorubicin^67^ induced the G2/M arrest in cancer cells, which can lead to the initiation of cell death.

### 3.5 Cytotoxicity Studies on Cell Spheroids

As 3D cancer cell culture models are mimicking *in vivo* cell behavior, these studies were focused on determining the cytotoxicity of doped cubosomes on such models. The spheroid response to the exposure of various synthesized compounds and different incubation times is presented in Figure 4S.

**Figure 10:**
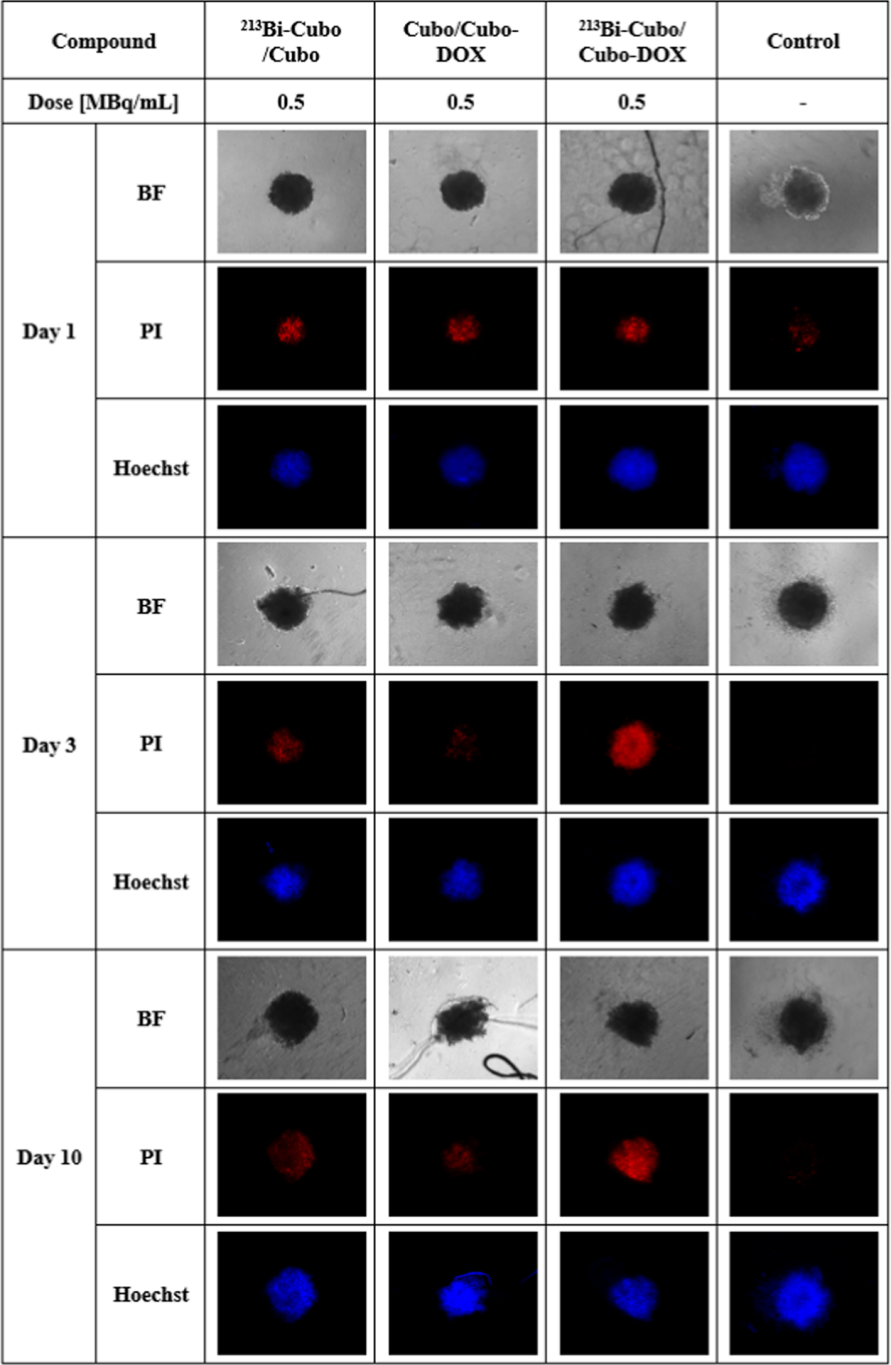
Bright-field (BF), propidium iodide (PI), and Hoechst 33258 (Hoechst) representative micrographs of HeLa spheroids treated with ^213^Bi-Cubo/Cubo, Cubo/DOX-Cubo, and ^213^Bi-Cubo/DOX-Cubo (500 kBq/mL of ^213^Bi; DOX concentration 0.2 μg/mL) and untreated (control) after 1, 3, and 10 days of incubation.

Additionally, to further verify the effect of the tested compounds on the 3D cancer cell culture models, they were subjected to propidium iodide (PI) and Hoechst 33258 (Hoechst) staining (Figure 10). Propidium iodide penetrates the damaged cell membrane, so by examining the integrity of the cell membrane, it can be determined that cells that glow red (Figure 10) are necrotic or in late apoptosis, whereas Hoechst 33258 (blue color) was used to stain only live cells. The best distinction of signal intensities was obtained for a dose of 0.5 MBq/mL; these images are depicted in Figure 10.

On the first day after the treatment, all spheroids exhibit similar red signal intensities (PI). After an incubation time of 3 days, a more intense signal of propidium iodide indicating necrotic or late apoptotic cells in spheroids exposed to ^213^Bi-Cubo/DOX-Cubo occurred. Contrary to the combined therapy, the treatment with only ^213^Bi-Cubo or DOX-Cubo showed a less intense red signal suggesting lower cytotoxicity. This overall efficacy trend was also observed after 10 days of incubation. These findings are comparable with the MTS assay and flow cytometry studies where the highest cytotoxicity and cell cycle block in the G2/M phase for sequential treatment were observed.

## 4 Conclusions

This work is the first *in vitro* study where α radionuclide and chemotherapeutic are encapsulated in cubosomes. The studies carried out here convince us that the cubosomes are useful carriers for both DOX and the ^213^Bi-DOTAGA-OA complex and may be promising in the combined radionuclide-chemotherapy. The addition of α emitter ^213^Bi to DOX will allow a significant reduction of the therapeutic dose of cardiotoxic DOX while maintaining the high effectiveness of the drug. However, it should also be taken into consideration that the presence of DOTAGA-OA in the carrier retards the release of DOX resulting in lower cytotoxicity, as shown by the release studies with spectrophotometric monitoring. Electrostatic attraction between the positively charged DOX (p*K*_a_ 8.4) and the negatively charged ligand is not unexpected leading to the slower release of the drug.

Therefore, we find it beneficial to deliver both drugs in separate liquid-crystalline carriers, DOX-Cubo, and ^213^Bi-Cubo to avoid molecular interactions between the positively charged DOX and negatively charged ligand complexing ^213^Bi. Sequential delivery of cubosomes, each with one drug, has been considered the best therapy for cancer cells.

In conclusion, a multimodal approach combining targeted radionuclide therapy with the administration of cytostatic drugs is a promising therapeutic concept that takes advantage of the synergistic effect of delivering both drugs encapsulated in separate carriers. Further studies should be directed toward the conjugation of the cubosomes with biomolecules targeting receptors overexpressed in tumor cells.

## Supporting Information Available

Final compositions of the cubic phases doped with different amounts of DOTAGA-OA (Table 1S); phase parameter calculation based on SAXS measurements (1S); redox process of DOX (Scheme 1S); representative differential pulse voltammograms for DOX incorporated into mesophase with or without DOTAGA-OA dopant (Figure 1S); representative graphs of the flow cytometry analysis after the treatment of HeLa cells with ^213^Bi-Cubo/DOX-Cubo and with no treatment (Figure 2S); representative histograms of cell cycle analysis of HeLa cells after treatment with ^213^Bi-Cubo/DOX-Cubo and without the treatment (Figure 3S); and microscope representative images treated with ^213^Bi-Cubo/Cubo, Cubo/DOX-Cubo, and ^213^Bi-Cubo/DOX-Cubo and untreated HeLa spheroids (Figure 4S) (PDF)
