# Supplementary material for: Cubosomal Lipid Formulation for Combination Cancer Treatment: Delivery of a Chemotherapeutic Agent and Complexed α-Particle Emitter 213Bi
Source: Mol Pharm. 2022 Jul 18;19(8):2818–31. doi: 10.1021/acs.molpharmaceut.2c00182 (PMC9346610; doi:10.1021/acs.molpharmaceut.2c00182)
Supplement: Supplementary file 1 — mp2c00182_si_001.pdf [file mp2c00182_si_001.pdf]

Supporting Information

Cubosomal Lipid Formulation for combination cancer treatment: delivery of chemotherapeutic agent and complexed α-particle emitter ^{213}Bi

Adrianna Cytryniak[a], Kinga Żelechowska-Matysiak[b], Ewa Nazaruk[a], Renata Bilewicz[a], Rafał Walczak[b], Emilia Majka[b], Adam Mames[c], Frank Bruchertseifer[d], Alfred Morgenstern[d], Aleksander Bilewicz[b], Agnieszka Majkowska-Pilip[b]*

[a]*Faculty of Chemistry, University of Warsaw, Pasteura 1, 02-093 Warsaw, Poland*

[b]*Centre of Radiochemistry and Nuclear Chemistry, Institute of Nuclear Chemistry and Technology, Dorodna 16, 03-195 Warsaw, Poland*

[c]*Institute of Physical Chemistry, Polish Academy of Sciences, Kasprzaka 44/52, 01-224 Warsaw, Poland*

[d]*Directorate for Nuclear Safety and Security, European Commission, Joint Research Centre, Postfach 2340, 76125 Karlsruhe, Germany.*

**Correspondence: a.majkowska@ichtj.waw.pl; Tel.: +48-22-504-1011*

Table 1S. The final compositions of the mesophases doped with DOTAGA-OA.

**Phase**	**Final composition**	**Components ratio [wt. %]**
0.5 % DOTAGA-OA	GMO/DOTAGA-OA/buffer	59.21/0.53/40.26
1.9 % DOTAGA-OA	GMO/DOTAGA-OA/buffer	58.3/1.90/39.80
3 % DOTAGA-OA	GMO/DOTAGA-OA/buffer	57.05/3.01/39.94
5.5 % DOTAGA-OA	GMO/DOTAGA-OA/buffer	54.59/5.50/39.91
7 % DOTAGA-OA	GMO/DOTAGA-OA/buffer	52.80/7.07/40.13
12 % DOTAGA-OA	GMO/DOTAGA-OA/buffer	46.56/12.01/34.43
15 % DOTAGA-OA	GMO/DOTAGA-OA/buffer	45.95/15.00/39.05

1S Phase parameters calculation based on SAXS measurements

Two-dimensional patterns were integrated into 1D scattering functions I(q) (where q (nm^{-1}) is the length of the scattering vector). The scattering vector q was determined from the scattering angle using the relationship $q = (4\pi/\lambda)\sin\theta$, where 2θ is the scattering angle, and λ is the wavelength of radiation. Before measurements, samples were loaded into 1.5 mm capillaries sealed with epoxy glue (UHU). Measurements were performed at 25 °C and 37 °C and

scattering intensities were collected over a period of 3 h for the dispersed systems. Equation (1) was used to calculate the lattice parameter (a):

$$a = \frac{2\pi}{q} \times \sqrt{h^2 + k^2 + l^2} \qquad (1)$$

where q is the scattering vector; h, k, l—Miller indices of the Bragg peak.

The aqueous channel radius (r_w) was obtained from the Equation (2):

$$r_w = \left(-\frac{A_0}{2\pi X}\right)^{\frac{1}{2}} a - l \qquad (2)$$

For the Pn3m phase, $A_0 = 1.919$, $\chi = -2$.We used the fixed value of lipid length (l) of 1.8 nm based on the literature data [1].

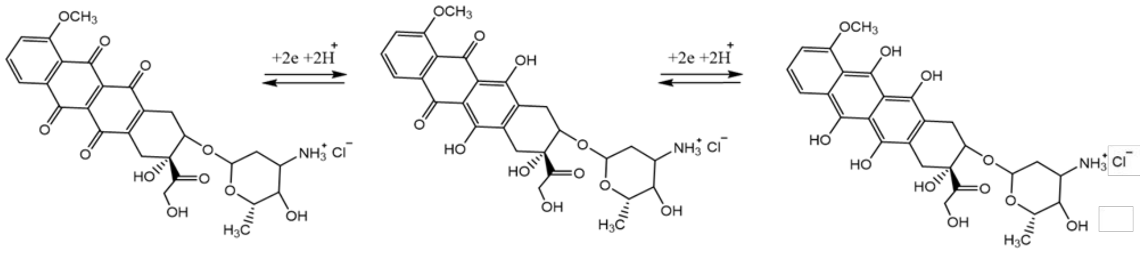

Scheme 1S. Redox Process of DOX.

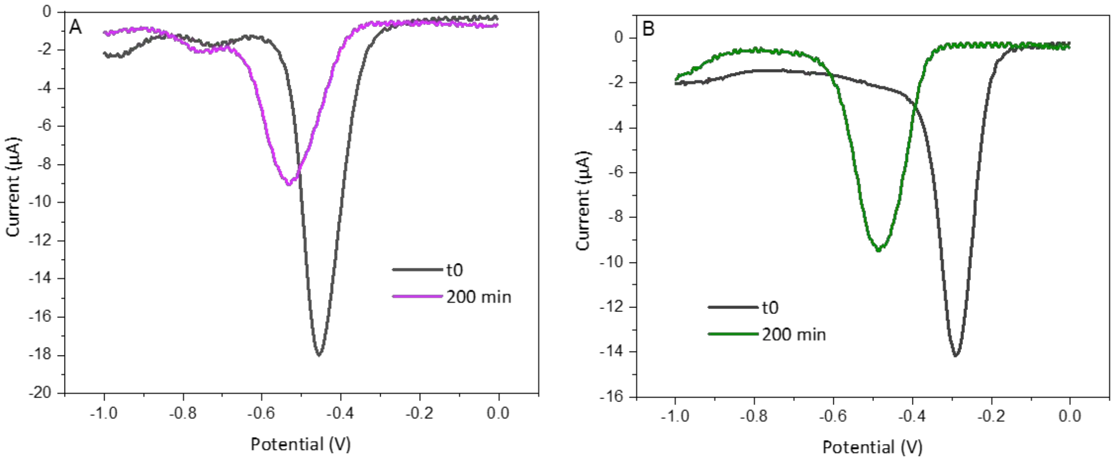

Figure 1S. DP voltammograms recorded on GCE at the moment of immersion of the electrodes in the buffer (t0) and after 200 min of DOX release form LCPs (A) without DOTAGA-OA and (B) doped with DOTAGA-OA ligand. Amplitude: ΔE=50 mV, pulse time: tp=50 ms.

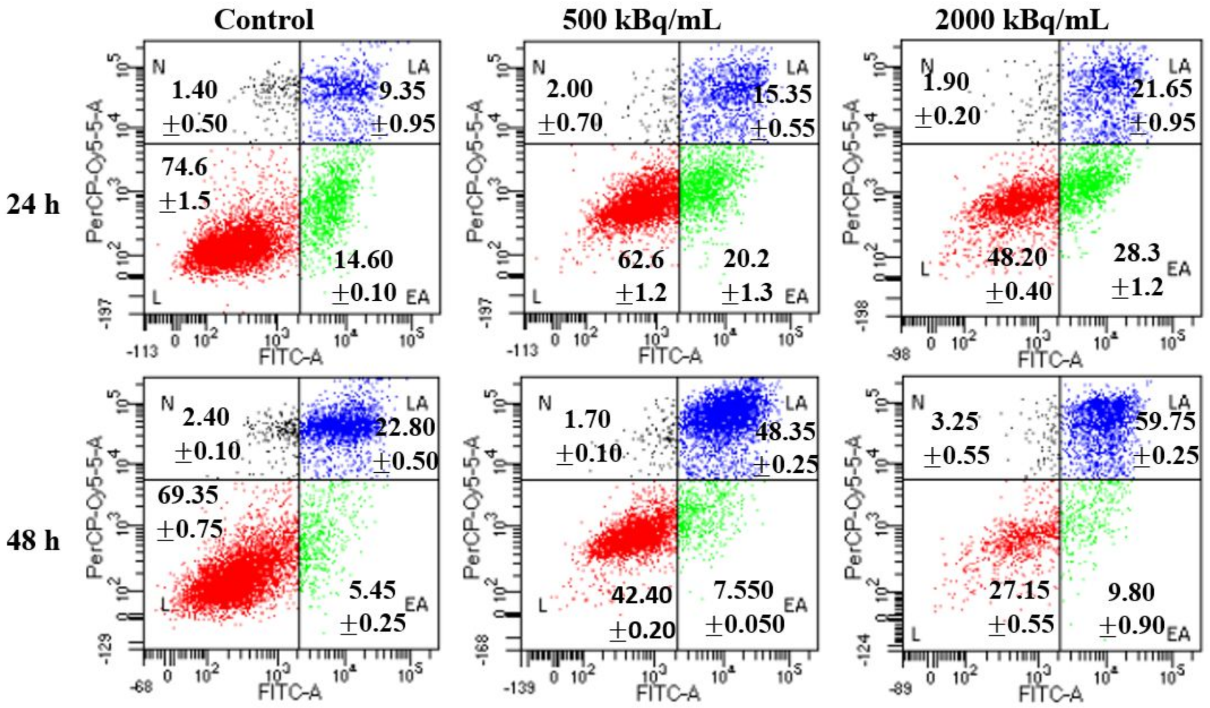

Figure 2S. Effect of sequential ^{213}Bi-Cubo/DOX-Cubo delivery on cell apoptosis. Representative graphs of the flow cytometry analysis after the treatment with 500 kBq/mL and 2000 kBq/mL of ^{213}Bi-Cubo/DOX-Cubo and with no treatment (control) for 24 h and 48 h. L-live cells, EA-early apoptosis, LA-late apoptosis, N-necrosis.

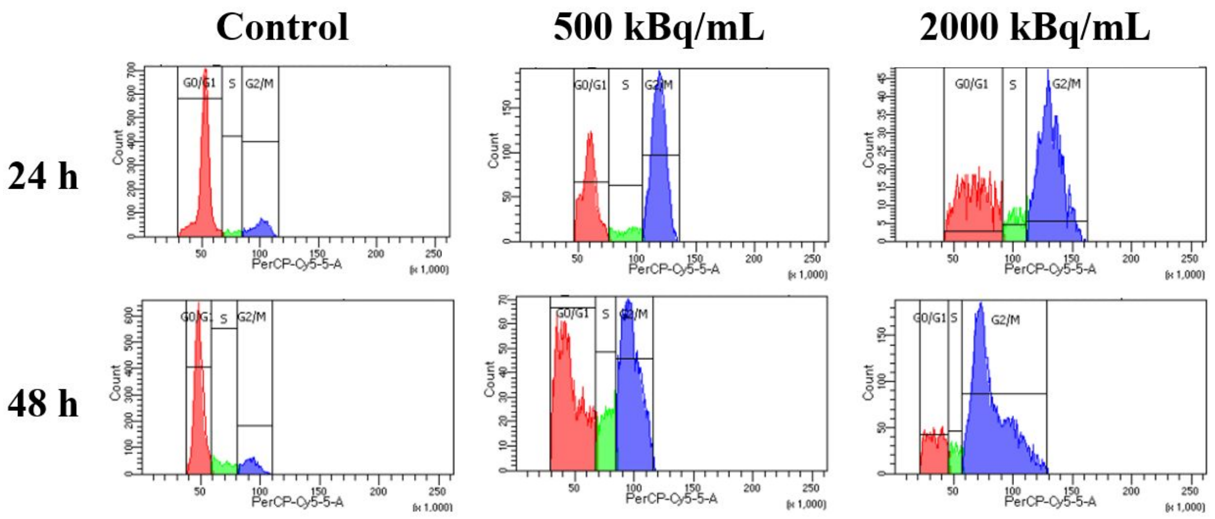

Figure 3S. Cell cycle analysis of HeLa cells after treatment with 500 kBq/mL and 2000 kBq/mL of ^{213}Bi-Cubo/DOX-Cubo and without the treatment (control) for 24 h and 48 h. Representative histograms showing the proportions of cells in G0/G1, S and G2/M phase.

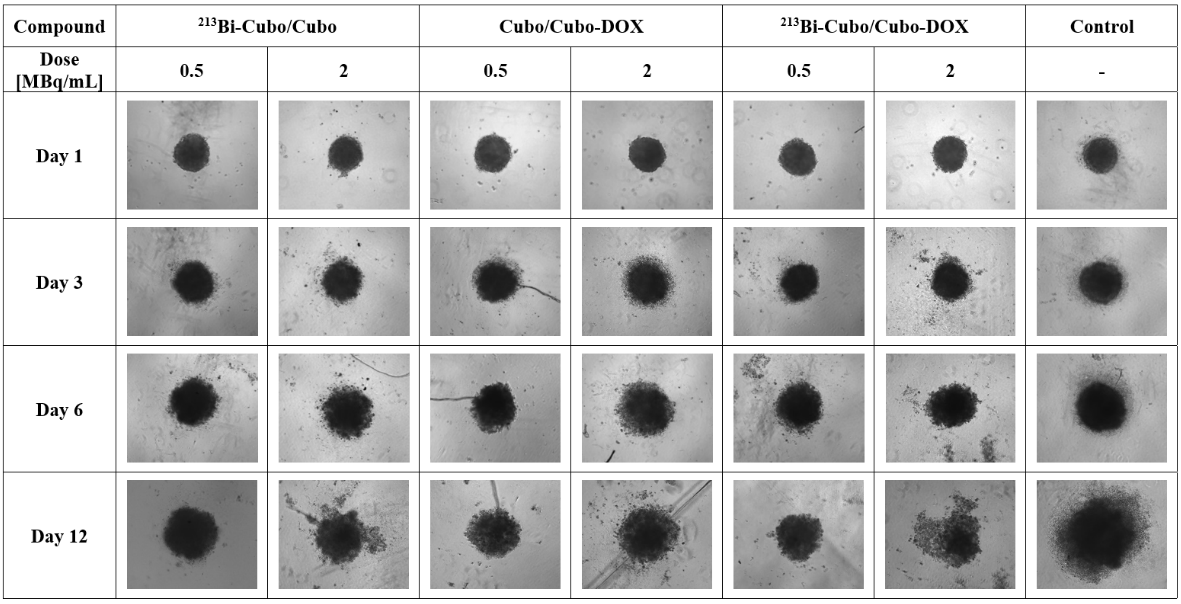

Figure 4S. Microscope representative images of treated with ^{213}Bi-Cubo/Cubo, Cubo/DOX-Cubo, ^{213}Bi-Cubo/DOX-Cubo (500 kBq/mL and 2000 kBq/mL of ^{213}Bi; DOX concentration 0.2 μg/mL) and untreated (control) HeLa spheroids after 1, 3, 6 and 12 days of incubation.

Significantly larger differences in the size and structure of the spheroids can be seen in the images from subsequent measurement days, where on day 12 all spheroids are meaningly smaller than the control. The results indicate that combined therapy with ^{213}Bi-Cubo/DOX-Cubo at a radioactivity level of 2000 kBq/mL significantly inhibits spheroid growth compared to their initial size. Contrary to this treatment, the same dose of only ^{213}Bi (2000 kBq/mL) or DOX (0.2 μg/mL) loaded in cubosomes has less influence on spheroid growth, as the size of treated spheroids is comparable to untreated spheroids.

References:

(1) Oka, T. Small-Angle X-Ray Crystallography on Single-Crystal Regions of Inverse Bicontinuous Cubic Phases: Lipid Bilayer Structures and Gaussian Curvature-Dependent Fluctuations. *J. Phys. Chem. B* **2017**, *121* (50), 11399–11409. https://doi.org/10.1021/acs.jpcb.7b08589.
